# Adaptability of Foxtail Millet Varieties Based on Photosynthetic Performance and Agronomic Traits

**DOI:** 10.3390/plants14162502

**Published:** 2025-08-12

**Authors:** Shulin Gao, Chenxu Wang, Xu Yang, Tianyu Ji, Suqi Shang, Shuo Li, Yinyuan Wen, Jianhong Ren, Xiaorui Li, Juan Zhao, Chunyan Hu, Xiangyang Yuan, Shuqi Dong

**Affiliations:** 1 College of Agriculture, Shanxi Agricultural University, Taigu 030800, China; gaoshulin1005@163.com (S.G.); once189396@163.com (C.W.); yyyangxuwww@126.com (X.Y.); echo180716@163.com (T.J.); ssq2025@163.com (S.S.); leesure2025@163.com (S.L.); wenyinyuan@126.com (Y.W.); lixiaorui@sxau.edu.cn (X.L.); sxndzhaojuan@163.com (J.Z.); 2 College of Life Sciences, Shanxi Agricultural University, Taigu 030800, China; renjh@sxau.edu.cn; 3 College of Plant Protection, Shanxi Agricultural University, Taigu 030800, China; hucy4216@163.com; 4 Special Orphan Crops Research Center of the Loess Plateau, MARA, Shanxi Agricultural University, Taigu 030800, China

**Keywords:** foxtail millet (*Setaria italica*), agronomic traits, variety adaptability, diurnal variation of photosynthesis, chlorophyll fluorescence parameters

## Abstract

As a strategic crop of dry farming in northern China, the photosynthetic characteristics and stress resistance of foxtail millet (*Setaria italica* L.) are crucial to yield formation. This study aimed to explore the physiological characteristics of various foxtail millet varieties and screen high-efficiency varieties adapted to semi-arid climates. In the agro-pastoral ecotone of northern Shanxi Province, the physiological and ecological parameters, etc. of six cultivars were measured. The results showed that different cultivars had bimodal diurnal photosynthetic curves with distinct peak values and midday depression degrees, reflecting varied responses to high midday temperature and light stress. Dabaigu and Jingu 21 performed superiorly, with mean daily net photosynthetic rates (Pn) of 22.99 and 20.72 µmol·m^−2^·s^−1^, significantly higher than Jinmiao K1 (12.87 µmol·m^−2^·s^−1^). Chlorophyll fluorescence analysis showed Dabaigu had higher potential activity (*F_v_*/*F*_0_) of 3.98 than Jinmiao K1 (2.40). Jingu 21 synergistically optimized plant height, stem diameter, and biomass accumulation. Dabaigu and Jingu 21 are elite cultivars for the agro-pastoral ecotone of northern Shanxi Province due to high photosynthetic efficiency, strong photoprotection, and morphological plasticity.

## 1. Introduction

Foxtail millet (*Setaria italica* L.) is a C_4_ crop with high economic and nutritional value [1,2]. It has been cultivated for more than 7000 years and is widely distributed in temperate and tropical regions of Eurasia [3]. As a crucial minor cereal crop, foxtail millet holds substantial agricultural importance due to its significant production value and adaptability [4]. In the arid and semi-arid regions of northern China, foxtail millet achieves annual grain yields ranging from 3.5 to 5 tonnes per hectare [5]. As a model C_4_ plant, foxtail millet exhibits distinctive physiological traits [6]. This crop demonstrates exceptional drought tolerance and high photosynthetic efficiency, making it an ideal candidate for sustainable agricultural systems in arid regions [7]. Consequently, addressing the adaptability of diverse foxtail millet varieties to varying environments is imperative for developing resilient cropping systems and to enhance yield and quality in the face of global climate change and rising food demand. Currently, scholars have estimated the potential breeding value of traits related to yield and grain quality through research on the foxtail millet genome, providing a pathway for breeding foxtail millet varieties adapted to climate change [8,9].

Light profoundly influences plant growth and development, physiological characteristics, biochemistry, and morphogenesis [10,11]. Photosynthesis is essential for plant growth and development, constituting a critical component of planetary life-support systems [12]. The primary engine of plant productivity is photosynthesis. Model simulations and proof-of-concept studies show that yield can be improved by enhancing photosynthetic efficiency [13]. The environmental and physiological factors influencing photosynthesis constitute a sophisticated regulatory network, and precise modulation of these factors can substantially boost photosynthetic capacity. In maize (*Zea mays* L.), optimized nitrogen fertilization regimes or modified cropping systems can effectively enhance leaf photosynthetic rates by improving leaf nitrogen content and canopy light interception efficiency [14]. Environmental factors exert multidimensional regulatory effects on photosynthesis in maize, where key variables such as atmospheric CO_2_ concentration and low temperature significantly alter photosynthetic performance [15,16]. Additionally, maize can further enhance photosynthetic efficiency through the synergistic integration of intrinsic physiological regulation mechanisms and morphological adaptation strategies. Research demonstrates that maize enhances CO_2_ fixation capacity and consequently improves photosynthetic efficiency by optimizing leaf anatomical structures and photosynthetic enzyme activity [17,18].

Systematic research on the photosynthetic mechanisms of C_4_ crops has accumulated over several years; for example, past, present, and future studies of C_4_ photosynthesis [19]. Furthermore, time-resolved transcriptome analysis confirmed that the expression of key C_4_ photosynthetic enzyme genes in maize (e.g., *PEPC* and *RBCS*) is tightly regulated by the circadian rhythm. Mesophyll cells and bundle sheath cells coordinately optimize carbon fixation efficiency during the light–dark cycle [20]. However, the unique carbon concentration mechanism and stress response adaptations of foxtail millet demand comprehensive mechanistic dissection. Multiple studies confirm that leaf photosynthetic rate directly governs dry matter accumulation in foxtail millet. This enhancement of biomass further regulates assimilate partitioning patterns. Consequently, photosynthetic efficiency ultimately determines the final yield outcomes [21]. For instance, under optimal irradiance and temperature regimes, elevated photosynthetic performance promotes efficient CO_2_ assimilation in foxtail millet leaves. This metabolic process generates abundant photosynthates, thereby supplying sufficient carbohydrates for growth demands across all plant organs. Stomata function as essential gateways for bidirectional gas exchange between plants and their environment. Their dynamic aperture adjustments precisely control CO_2_ availability for photosynthetic carboxylation while simultaneously governing water vapor efflux through transpiration. Stomatal regulation mediates the critical trade-off between photosynthetic efficiency and plant water balance [22]. For instance, under drought stress, sorghum (*Sorghum bicolor*) maintains elevated Pn and improved WUE through synergistic stomatal regulation and osmolyte accumulation. This involves diurnal stomatal closure to minimize water loss, coupled with biosynthesis of osmolytes such as proline and glycine betaine for cellular osmotic adjustment [23,24]. Functioning as real-time biosensors, chlorophyll fluorescence parameters simultaneously quantify PSII electron flux and track the activation kinetics of essential photoprotection subsystems, including energy dissipation via quenching and photosynthetic state transitions. Such comprehensive monitoring capability positions these parameters as fundamental tools for decoding light adaptation strategies in natural ecosystems [25]. High irradiance and thermal co-stress consistently induce photoinhibition, causing direct damage to PSII reaction centers [26]. Chlorophyll fluorescence parameters (e.g., *F_v_*/*F_m_*, *qN*) quantitatively assess photochemical efficiency and non-photochemical quenching capacity, thereby revealing interspecific divergence in light acclimation strategies [27].

Agronomic traits represent the morphological manifestation of photosynthetic performance. The coordination among plant height, stem diameter, and leaf area in foxtail millet collectively influences both its growth vigor and yield potential [28,29]. Increased plant height combined with expanded leaf area contributes to greater light interception capacity by the foliage, thereby enhancing photosynthetic efficiency. However, robust stem development is essential to provide mechanical stability and mitigate the risk of lodging.

Increasingly frequent extreme climate events, specifically drought, heatwaves, and torrential rainfall, pose severe threats to foxtail millet production under accelerating global climate change [30]. At present, comprehensive research specifically addressing the photosynthetic efficiency of foxtail millet within the agro-pastoral ecotone of northern Shanxi Province remains limited. Against this backdrop, undertaking comprehensive investigations into the photosynthetic physiology and agronomic performance of distinct foxtail millet varieties under complex and variable environmental conditions is essential. Therefore, the identification and development of novel foxtail millet varieties showing enhanced stress tolerance, high yield potential, and superior grain quality represent a critical challenge that demands urgent resolution within the current scientific research domain of this crop. Furthermore, elucidating the intrinsic relationships and coordinated regulatory mechanisms between photosynthetic physiology and agronomic traits has significant theoretical and practical implications for optimizing foxtail millet cultivation management, enhancing resource use efficiency, and advancing the sustainable development of the foxtail millet industry. The synergistic regulation mechanism of photosynthetic physiology and agronomic traits analyzed in this study can enrich the theoretical system of stress resistance physiology and high-yield cultivation of foxtail millet, and provide new ideas and directions for research in related fields. In response to global climate change, the results of this study will help improve the ability of foxtail millet production in the agro-pastoral ecotone of northern Shanxi to cope with extreme climate events, ensure regional food security, and promote the sustainable development of the foxtail millet industry.

## 2. Results

### 2.1. Comparison of Physiological and Ecological Parameters of Different Foxtail Millet Varieties

#### 2.1.1. Comparison of Diurnal Variation in Net Photosynthetic Rate

**Figure 1:**
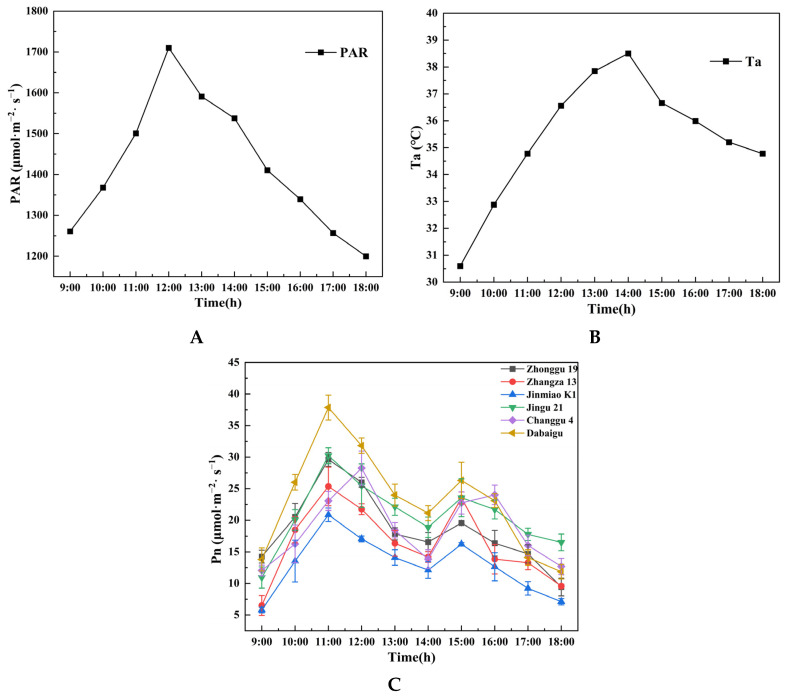
Diurnal variations in (**A**) photosynthetically active radiation (PAR), (**B**) ambient temperature (Ta), and (**C**) net photosynthetic rate (Pn) of different foxtail millet cultivars during daytime (9:00–18:00).

Figure 1A shows a rapid increase in photosynthetically active radiation (PAR) from 9:00 to 12:00, reaching a peak irradiance of 1710 µmol·m^−2^·s^−1^ before undergoing gradual attenuation during the afternoon. This diurnal pattern aligns with established solar radiation dynamics under clear sky conditions as reported in prior research.

Figure 1B shows that ambient temperature progressively increased from 09:00, reaching its diurnal maximum of 38.2 °C at 14:00 before undergoing subsequent cooling.

Figure 1C shows a bimodal diurnal pattern in Pn across foxtail millet varieties. During the morning hours, Pn across foxtail millet cultivars shows a progressive ascent in response to increasing PAR and ambient temperature (Ta), culminating in an initial peak between 11:00 and 12:00. The cultivar Dabaigu achieves the maximum Pn amplitude of 40 µmol·m^−2^·s^−1^ in this diurnal phase. At midday, plants exhibit photosynthetic depression characterized by reduced Pn. This primarily results from stomatal closure and impaired PSII activity driven by excessive PAR and high ambient temperatures. Around 15:00, specific foxtail millet cultivars develop a secondary Pn peak responding to moderate PAR and temperature, yet this peak consistently remains below the initial maximum. Subsequently, photosynthetic rates decline progressively in response to decreasing irradiance and temperature.

#### 2.1.2. Comparison of Additional Parameters

**Figure 2:**
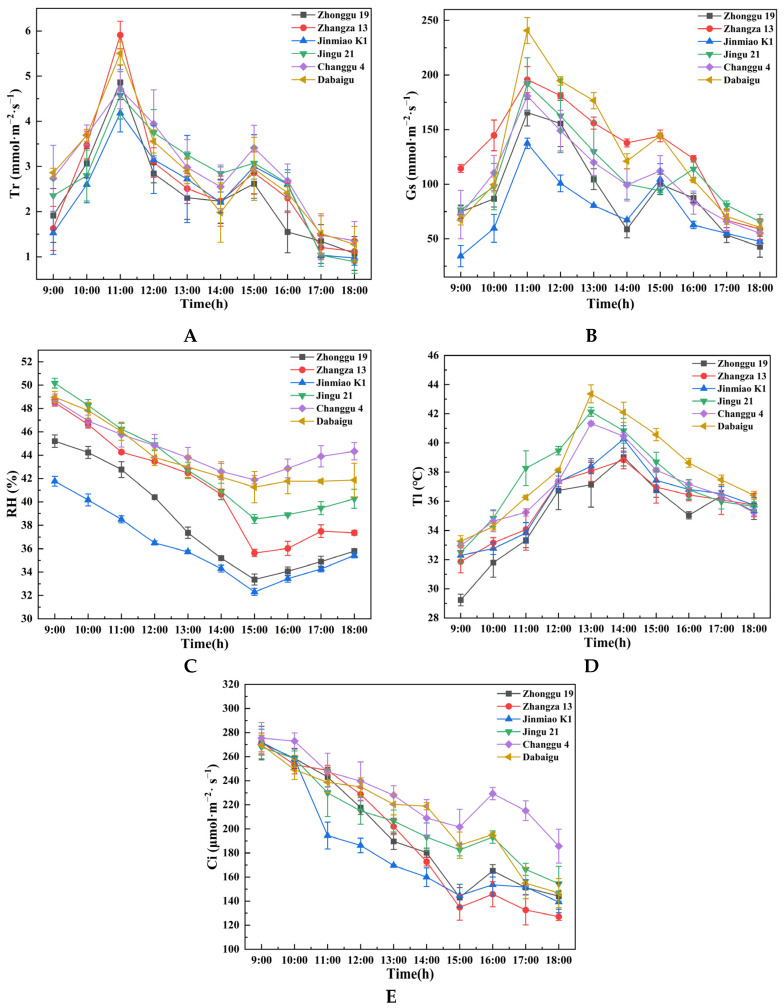
Diurnal variations in (**A**) transpiration rate (Tr), (**B**) stomatal conductance (Gs), (**C**) relative air humidity (RH), (**D**) leaf temperature (Tl), and (**E**) intercellular CO_2_ concentration (Ci). Error bars indicate standard errors, illustrating cultivar-specific differences in transpiration-related traits during daytime measurements (9:00–18:00).

Figure 2A shows bimodal diurnal transpiration patterns across all foxtail millet cultivars. Morning transpiration increased progressively, with rising irradiance and temperature peaking initially near 11:00. Subsequently, midday stomatal closure and photosynthetic midday depression caused by high temperature or light intensity led to a reduction in transpiration. A secondary peak emerged in select cultivars around 15:00, consistently lower than the initial maximum, followed by a progressive decline with changing environmental parameters. Although the diurnal variation trend of transpiration rate among the different varieties is the same, there are some differences in the value. Zhangza 13 performed best at noon, and it first reached the first peak value (about 6 mmol·m^−2^·s^−1^). However, the first peak value of Jinmiao K1 was significantly lower than that of other varieties of foxtail millet.

Figure 2B shows analogous diurnal patterns in stomatal conductance (Gs) and transpiration rate (Tr) among foxtail millet cultivars, predominantly displaying bimodal curves. Dabaigu still performed best (about 250 mmol·m^−2^·s^−1^).

The relative air humidity (RH) showed a downward trend as a whole. At about 15:00, it reached a trough, and some varieties were as low as about 24% (Figure 2C).

The leaf temperature (Tl) showed a trend of increasing first and then decreasing during the day, reaching the highest at about 14:00, with Dabaigu and Jingu 21 having up to about 43 °C; in Zhangza 13 and Zhonggu 19, the highest temperature is only about 39 °C (Figure 2D).

Figure 2E shows intricate diurnal dynamics in intercellular CO_2_ concentration (Ci) among foxtail millet cultivars. Elevated Ci levels during the early photoperiod progressively depleted as photosynthetic carbon assimilation intensified. Midday photosynthetic depression coupled with stomatal adjustments elevated Ci in Dabaigu and Zhonggu 19.

#### 2.1.3. Comparison of Leaf Water Use Efficiency

WUE differed among foxtail millet cultivars (Table 1). Jingu 21 and Dabaigu show significantly higher mean Pn (20.72 ± 0.31 and 22.99 ± 0.23 µmol·m^−2^·s^−1^, respectively) than other foxtail millet cultivars. Changgu 4 and Dabaigu show higher mean Tr at 2.95 ± 0.16 and 2.86 ± 0.03 µmol·m^−2^·s^−1^, respectively, than other foxtail millet cultivars. Dabaigu shows the highest WUE (8.04 ± 0.05 µmol CO_2_/mmol H_2_O), while Jinmiao K1 shows the lowest (5.36 ± 0.15 µmol CO_2_/mmol H_2_O).

#### 2.1.4. Comparison of Mean Diurnal Variation in Ecophysiological Parameters

Changgu 4 exhibits a significantly higher mean daily Ci (230.42 ± 4.12 µmol·m^−2^·s^−1^) than Jinmiao K1 (182.95 ± 1.70 µmol·m^−2^·s^−1^). The reduced Ci in Jinmiao K1 may limit the dark reactions of photosynthesis, consequently impairing photosynthetic efficiency. Zhangza 13 shows a significantly higher mean daily Gs (132.33 ± 3.01 mmol·m^−2^·s^−1^) than Jinmiao K1 (74.96 ± 1.83 mmol·m^−2^·s^−1^). This genotypic variation in stomatal aperture regulates CO_2_ uptake and transpiration. The elevated Gs in Zhangza 13 enhances CO_2_ diffusion into the leaves, providing sufficient substrate for photosynthesis and potentially increasing photosynthetic rates. However, elevated Gs accelerates stomatal water loss, potentially reducing WUE. Compared with Zhangza 13, the lower mean daily Gs in Jinmiao K1 restricts CO_2_ uptake, impairing photosynthesis while potentially conferring water conservation advantages by reducing excessive water loss. Significant differences (*p* < 0.05) occurred in mean daily RH among various foxtail millet cultivars, ranging from 33.38% to 38.33%. Mean daily Tl ranged from 35.08 °C to 38.08 °C across foxtail millet cultivars, with no significant intervarietal differences (*p* > 0.05) (Table 2).

### 2.2. Comparison of Chlorophyll Fluorescence Kinetics and Chlorophyll Content

**Figure 3:**
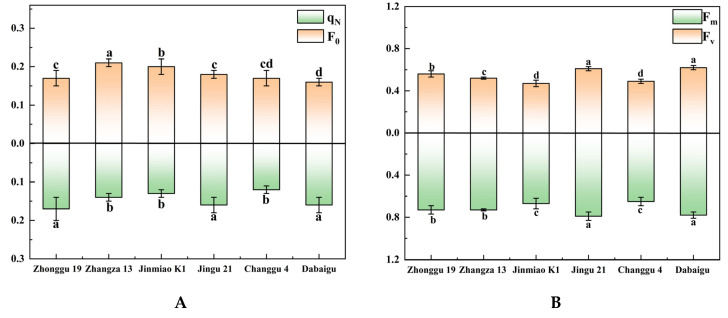

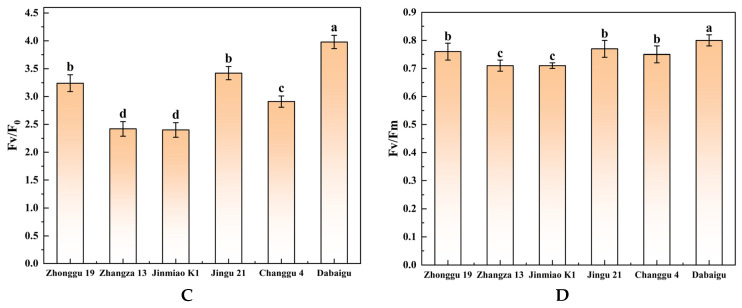
Comparison of chlorophyll fluorescence kinetic parameters of different foxtail millet cultivars. (**A**) Non-photochemical quenching (*qN*) and initial fluorescence (*F*_0_); (**B**) maximum fluorescence (*F_m_*) and variable fluorescence (*F_v_*); (**C**) potential activity (*F_v_*/*F*_0_); (**D**) maximum photochemical efficiency (*F_v_*/*F_m_*). Different lowercase letters indicate significant differences between cultivars at the *p* < 0.05 level (Duncan’s multiple comparison method based on one-way ANOVA).

Figure 3A–D show genotypic variation in chlorophyll fluorescence parameters among foxtail millet cultivars. Figure 3A shows significant inter-cultivar differences in *qN* and *F*_0_, with Zhangza 13 showing notably higher *F*_0_ values (about 0.21) than other cultivars, such as Dabaigu (about 0.16). Jinmiao K1 and Changgu 4 showed relatively lower values in both parameters: *F_m_* for Changgu 4 was about 0.65, significantly lower than that of Jingu 21 (about 0.79), while Jinmiao K1 exhibited the lowest *F_v_* (about 0.47) among all cultivars (Figure 3B). Figure 3C,D similarly exhibit significant inter-cultivar differences, with Jinmiao K1 and Zhangza 13 showing lower values than most cultivars in both parameters. This demonstrates the structural and functional divergence in PSII among foxtail millet cultivars. Specifically, Jinmiao K1’s *F_v_*/*F*_0_ (about 2.40) was lower than all tested varieties, and its *F_v_*/*F_m_* (about 0.71) was notably lower than that of Dabaigu (about 0.80). These findings collectively indicate that these two cultivars have a reduced ability to channel absorbed light energy into photochemical reactions, possibly due to differences in PSII repair mechanisms or antioxidant defense systems under the prevailing field conditions.

**Figure 4:**
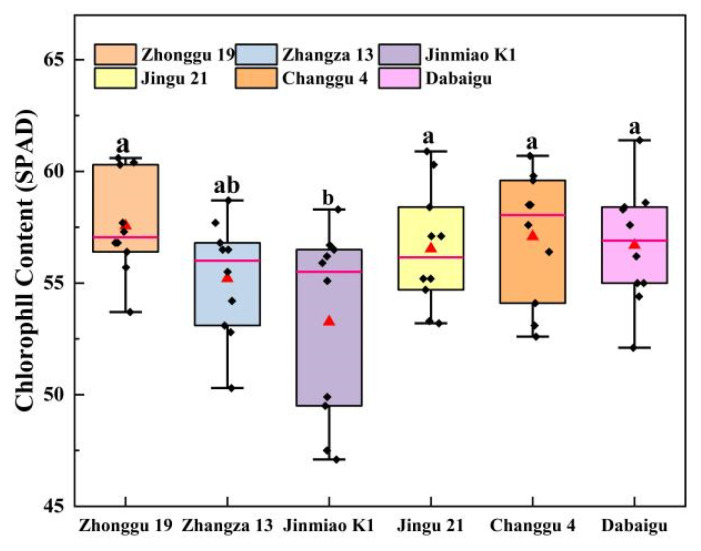
Chlorophyll content of different cultivars of foxtail millet leaves. Different lowercase letters indicate significant differences between cultivars at the *p* < 0.05 level (Duncan’s multiple comparison method based on one-way ANOVA).

Figure 4 indicates no significant differences in chlorophyll content (*p* > 0.05) among Zhonggu 19, Jingu 21, Changgu 4, and Dabaigu, but significant differences compared with Zhangza 13 and Jinmiao K1. Overall, chlorophyll content across foxtail millet cultivars follows the descending order Dabaigu > Changgu 4 > Zhonggu 19 > Jingu 21 > Zhangza 13 > Jinmiao K1.

### 2.3. Comparison of Agronomic Traits

**Figure 5:**
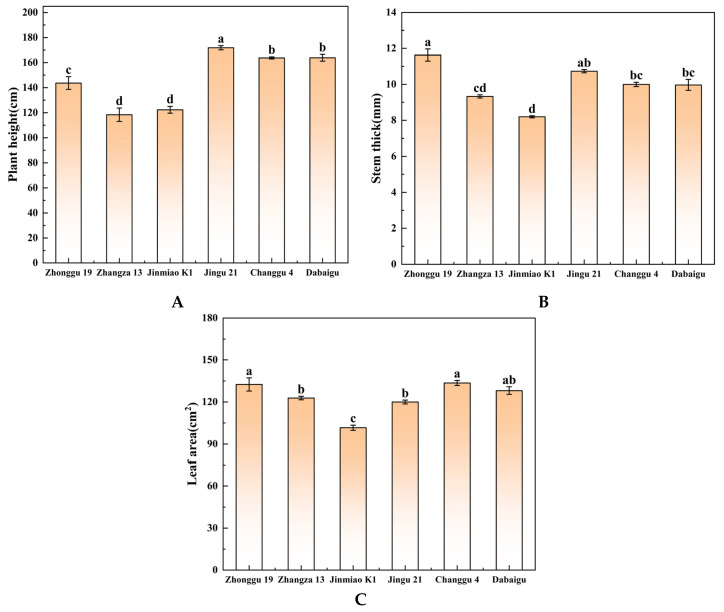
Different foxtail millet cultivars were measured for (**A**) plant height, (**B**) stem dimension, and (**C**) leaf area. Different lowercase letters indicate significant differences between cultivars at the *p* < 0.05 level (Duncan’s multiple comparison method based on one-way ANOVA).

Figure 5A–C show statistically significant differences in plant height, stem diameter, and leaf area among foxtail millet cultivars. Jingu 21 shows significantly greater plant height (about 170 cm) relative to other foxtail millet cultivars. Zhangza 13 and Jinmiao K1 exhibit significantly lower plant heights (about 115 cm) than other cultivars. Zhonggu 19 exhibits significantly greater stem diameter (about 11.5 mm) than other cultivars, whereas Jinmiao K1 exhibits significantly smaller stem diameter (about 8 mm). Zhonggu 19 and Changgu 4 exhibits significantly greater leaf area (about 130 cm^2^ and 135 cm^2^) versus Jinmiao K1 (about 100 cm^2^).

### 2.4. Correlation Analysis

**Figure 6:**
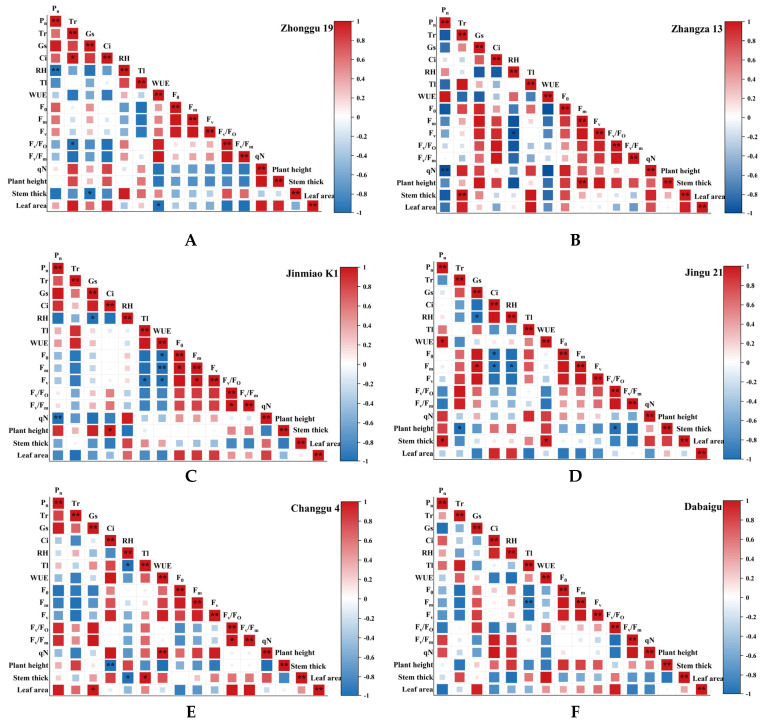
Comparison of the correlation between the parameters of different cultivars of foxtail millet. (**A**) Correlation analysis of Zhonggu 19; (**B**) Correlation analysis of Zhangza 13; (**C**) Correlation analysis of Jinmiao K1; (**D**) Correlation analysis of Jingu 21; (**E**) Correlation analysis of Changgu 4; (**F**) Correlation analysis of Dabaigu. Pearson correlation analysis was performed. Significance levels are marked as *p* < 0.05 (*) and *p* < 0.01 (**). Abbreviations: Pn, net photosynthetic rate; Tr, transpiration rate; Gs, stomatal conductance; Ci, intercellular CO_2_ concentration; RH, relative humidity; Tl, leaf temperature; WUE, leaf water use efficiency; *F*_0_, initial fluorescence; *F_m_*, maximum fluorescence; *F_v_*, variable fluorescence; *F_v_*/*F*_0_, potential activity; *F_v_*/*F_m_*, maximum photochemical efficiency; *qN*, non-photochemical quenching. Note: Red indicates positive correlations, blue indicates negative correlations, the depth of color represents the strength of the correlation degree, and the color bar on the right side from -1 to 1 indicates the range of the correlation coefficient.

Figure 6 shows significant positive correlations between Pn and Tr across foxtail millet cultivars. Gs also generally exhibits significant positive correlations with both Pn and Tr, though with cultivar-dependent variation in its strength. PSII parameters (*F_v_*/*F_m_*, *F_v_*/*F*_0_) exhibit differential correlations with other photosynthetic and agronomic traits across cultivars. For instance, *F_v_*/*F_m_* shows significant correlations with plant height and stem diameter in certain cultivars (e.g., Zhonggu 19 demonstrates significant positive correlations with plant height). WUE exhibits cultivar-specific correlation patterns with other photosynthetic parameters across foxtail millet cultivars. In certain foxtail millet cultivars, WUE positively correlates with Pn (e.g., Jingu 21). Select foxtail millet accessions demonstrate inverse correlative trends between WUE and Tr. Significant positive correlations between plant height and stem diameter occur across foxtail millet cultivars (e.g., Zhangza 13; *p* < 0.001), indicating coordinated vertical–radial growth patterns. Significant associations were also observed between agronomic traits and photosynthetic physiological parameters across different foxtail millet cultivars. As evidenced in some cultivars (e.g., Changgu 4), leaf area exhibits a positive correlation with the Pn. This relationship potentially arises because a larger leaf area provides an increased photosynthetic surface area, thereby enhancing the overall photosynthetic rate.

**Figure 7:**
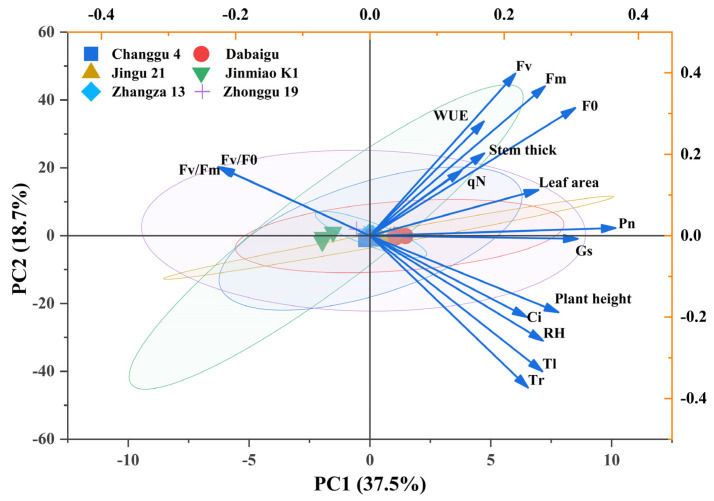
Principal component analysis among parameters of different cultivars of foxtail millet.

The first and second principal components collectively accounted for 56.2% of the total variation observed across 17 traits encompassing WUE, photosynthetic performance, and morphological characteristics in the studied foxtail millet cultivars. Comprehensive analysis reveals that the first four principal components exhibit eigenvalues exceeding 1, collectively capturing 83% of the total variance. The first principal component alone accounted for 37.5% of this variation, while the second principal component contributed 18.7% (Table 3). Within the first principal component, Pn and plant height emerged as the primary contributing traits. The second principal component was predominantly characterized by substantial contributions from WUE and Tr. PCA showed the characteristics of variety grouping: Zhangza 13 was significantly biased towards the positive axis of PC1 and the central axis of PC2, which was strongly associated with high photosynthetic and large leaf traits such as Pn, Gs, and leaf area, showing high photosynthetic growth type differentiation. Changgu 4 was biased to the negative axis of PC1 and the positive axis of PC2, and was significantly restricted by stomata due to its proximity to Ls and *F_v_*/*F*_0_, showing a stomatal-limited local separation. The confidence ellipses of Dabaigu, Jingu 21, Jinmiao K1, and Zhonggu 19 are highly overlapped and concentrated near the PC1 axis + PC2 axis. These varieties had a high degree of overlap in *F_v_*/*F_m_*, RH, plant height, and other trait vectors, indicating that they had little difference in photosynthetic system stability, plant type consistency, and environmental humidity adaptation. The contributions of various physiological and morphological indices (e.g., *F_v_*/*F*_0_, Gs, leaf area) to the target traits exhibited significant heterogeneity. Morphological indicators (e.g., plant height, stem diameter) and physiological parameters (WUE) synergistically explained 37.5% of the variation captured by the first principal component (PC1) across diverse foxtail millet cultivars (Figure 7).

## 3. Discussion

This study conducted a systematic comparison of photosynthetic physiological parameters and agronomic traits across six foxtail millet cultivars from the agro-pastoral ecotone of northern Shanxi Province. This investigation revealed significant variations in photosynthetic efficiency, stomatal regulation, light energy utilization, and morphological characteristics across the foxtail millet cultivars. The results demonstrated that the cultivars Dabaigu and Jingu 21 showed superior performance in key photosynthetic metrics, including Pn, chlorophyll content, and photochemical efficiency. However, Jinmiao K1 and Zhangza 13 showed inferior performance across multiple parameters. These findings deliver critical breeding references for foxtail millet cultivars in the agro-pastoral ecotone of northern Shanxi Province while providing robust scientific evidence for deciphering the intrinsic mechanisms governing photosynthetic physiology and environmental adaptation in this crop.

### 3.1. Diurnal Variation of Photosynthesis and Cultivar Specificity

This study observed consistent bimodal diurnal patterns in Pn across all foxtail millet cultivars, with a significantly higher magnitude at the first peak compared with the second. Specifically, Pn in all foxtail millet cultivars increased with rising PAR during the morning hours followed by midday photosynthetic depression under elevated temperatures and high irradiance, with some cultivars showing a secondary peak in the afternoon.

Extensive investigations by numerous scholars have documented diurnal photosynthetic patterns across diverse crop species within plant photosynthetic physiology research. Scholars have analyzed the daily process of photosynthesis in maize. The results showed that the diurnal variation of Pn in maize leaves showed a double-peak curve with a decrease at noon and an increase in the afternoon. There was a significant difference in the degree of midday depression of photosynthesis among maize varieties [31]. Furthermore, in-depth investigations into the regulatory mechanisms of stomatal anatomical traits on conductance dynamics have demonstrated that manipulating both the physical and physiological properties of guard cells can achieve dynamic synchronization between stomatal movement and mesophyll CO_2_ demand [32]. This approach not only significantly enhances water use efficiency but also maintains photosynthetic carbon fixation capacity. These findings provide critical scientific insights into the environmental adaptation mechanisms of plants’ photosynthetic physiology and offer valuable implications for genetic improvement of crops under climate change scenarios.

Foxtail millet shares conserved photosynthetic responses with maize and wheat under midday high irradiance and high temperature stress, universally showing susceptibility to photosynthetic depression [33,34]. This photosynthetic depression may arise from midday stomatal closure or reduced PSII activity under high irradiance and high temperature stress. Notably, Dabaigu and Jingu 21 showed significantly higher peak Pn than Jinmiao K1. This difference may be related to non-stomatal factors such as enzyme activity and light reaction efficiency (Table 1). Compared with other foxtail millet cultivars, Jingu 21 achieves synergistic optimization of photosynthesis and water conservation through balanced Gs (111.30 ± 8.30 mmol·m^−2^·s^−1^) and WUE (7.62 ± 0.62 µmol CO_2_/mmol H_2_O), demonstrating adaptive advantages in arid environments. Furthermore, the duration of photosynthetic depression and recovery capacity demonstrated cultivar-specific divergence, where Changgu 4 showed limited afternoon Pn resurgence, while Zhangza 13 displayed a more pronounced secondary peak. This divergence likely resulted from cultivar-dependent differences in photoprotective mechanisms including *qN* and PSII repair efficiency [35,36]. For the chlorophyll fluorescence parameters, Dabaigu showed significantly higher *F_v_*/*F_m_* (0.80) than Jinmiao K1 (0.71), indicating superior PSII photochemical efficiency, potentially through self-coordinated alleviation of photoinhibition. It has been demonstrated that variation in the steady-state CO_2_ assimilation rate (*ACS1*/*GAD1*) among maize cultivars closely correlated with the mesophyll to bundle sheath cell area ratio, whereas CO_2_ loss (Closs) under dynamic light was regulated by stomatal conductance kinetics and expression of ethylene synthesis genes [37]. Research revealed, through genome-wide association analysis, that variation in stomatal closing velocity among rice accessions correlates with NHX2 haplotypes, which encode a sodium/hydrogen exchanger regulating turgor pressure in guard cells. Haplotype III materials originating from arid regions concurrently showed enhanced WUE [38]. These findings suggest that inter-cultivar photosynthetic performance divergence may extend beyond stomatal regulation to involve molecular control mechanisms governing light energy harvesting and carbon assimilation pathways.

### 3.2. Coordination Between Stomatal Behavior and Water Use

Functioning as the central nexus linking photosynthesis and transpiration, stomatal dynamics directly regulate plant carbon–water exchange efficiency [39]. Our data revealed a highly significant positive correlation between Gs and Tr (*p* < 0.01), whereas WUE showed no statistically significant variation among cultivars (*p* > 0.05). This phenomenon reveals a classic physiological trade-off mechanism: cultivars with elevated stomatal conductance like Zhangza 13 significantly enhance Pn through increased CO_2_ influx yet incur pronounced water deficit due to heightened Tr. Conversely, cultivars with reduced stomatal conductance like Jinmiao K1 minimize water loss but constrain photosynthetic efficiency by restricting CO_2_ diffusion into the leaf tissues. This stomata–water homeostasis becomes particularly critical in arid habitats [40].

Notably, Jingu 21 and Dabaigu achieve a synergistic optimization of Gs and Tr. This physiological coordination allows these cultivars to maintain high photosynthetic efficiency under varying conditions without incurring a significant penalty in WUE. This represents a valuable adaptive trait, particularly in environments where water availability may be limiting or unpredictable. The underlying regulatory mechanism enabling this balance potentially involves complementary adaptive modifications at both the structural and biochemical levels. Its regulatory mechanism potentially involves adaptive modifications in leaf anatomy such as reduced stomatal density or enhanced mesophyll conductance, alongside fine-tuned hormonal signaling pathways exemplified by ABA-mediated delayed stomatal closure [41]. Subsequent studies should integrate microstructural analyses (e.g., stomatal density quantification) with hormonal metabolic profiling, including key gene expression in ABA-mediated signaling pathways, to precisely unravel the molecular physiological mechanisms underlying this synergistic regulation.

### 3.3. Synergistic Effects of Morphological Traits and Photosynthetic Performance

Agronomic trait analysis provided clear evidence of performance differentials among the studied foxtail millet cultivars. Specifically, Jingu 21 and Changgu 4 demonstrated statistically significant superiority over Jinmiao K1 and Zhangza 13 across several key morphological indicators that are fundamental to plant architecture and productivity. These included plant height, stem diameter, and leaf area index. From an ecological and physiological perspective, taller plant stature combined with greater leaf area generally confers a distinct advantage in terms of light interception capacity within a canopy, potentially leading to higher photosynthetic rates and biomass production [42]. However, this strategy necessitates a critical balance, as excessive height or foliage without proportional structural reinforcement can compromise stem mechanical strength, significantly increasing susceptibility to lodging (stem bending or breakage), which is a major cause of yield loss in cereal crops.

This research specifically illuminated a strong, positive correlation between stem diameter and plant height within Jingu 21. This observed morphological synergy suggests that Jingu 21 has undergone coordinated developmental optimization, effectively achieving a synergistic equilibrium between the competing demands of vigorous biomass accumulation through enhanced light capture and the essential requirement for robust physical stress resistance provided by thicker stems.

Conversely, Jinmiao K1 showed a contrasting phenotype characterized by significantly reduced plant height and notably slender stems relative to the other cultivars evaluated. This specific morphological configuration, while potentially offering inherent advantages in terms of reduced resource investment per stem unit, likely imposes constraints on its competitive ability for light interception within dense stands. The diminished stature restricts its positioning within the light gradient of the canopy, and the slender stems may limit the vascular capacity for resource transport. Consequently, these traits may act as physiological bottlenecks, ultimately limiting the plant’s capacity for photosynthate accumulation and partitioning to developing grains. Further correlation analysis substantiated these morphological relationships across the cultivars, revealing a highly significant positive correlation between plant height and stem diameter (*p* < 0.05). This strong interdependence underscores the general tendency for taller plants to develop thicker supporting structures. In contrast, the relationship between leaf area and Pn was found to be weaker and statistically non-significant (*p* > 0.05), indicating that simply possessing larger leaves does not uniformly guarantee proportionally higher photosynthetic output across all genetic backgrounds.

These divergent correlation patterns strongly suggest the existence of distinct resource allocation strategies employed by different foxtail millet cultivars as adaptive specializations. Jingu 21 appears to prioritize resource investment into stem development and strengthening, thereby enhancing its lodging resistance crucial trait for stability and yield protection, particularly under challenging environmental conditions like high winds or heavy rain. On the other hand, Changgu 4 directs a larger share of its resources towards expanding leaf area, a strategy primarily aimed at maximizing light energy capture efficiency to fuel growth and productivity, as visually supported by the data trends in Figure 6E [43]. This phenotypic plasticity serves as a crucial adaptive mechanism for plants responding to environmental fluctuations, reflecting synergistic optimization between growth strategies and stress resistance across cultivars.

### 3.4. Linkage Between Chlorophyll Fluorescence Kinetics and Photoprotective Mechanisms

Chlorophyll fluorescence kinetics serve as critical indicators for assessing photosystem performance in plants. This study revealed that Dabaigu showed significantly higher *F_m_* and *F_v_* than other cultivars, indicating superior light energy capture efficiency in its PSII reaction centers (Figure 3). Its elevated *F_v_*/*F_m_* ratio approaches the theoretical maximum for C_4_ crops [44]. It reflects exceptional efficiency in photochemical processes. In contrast to Dabaigu, Jinmiao K1 demonstrates a depressed *F_v_*/*F_m_* ratio, indicative of compromised light energy conversion efficiency in PSII, potentially attributable to photoinhibition or disruptions in the electron transport chain.

Notably, Dabaigu showed significantly elevated *F*_0_ compared with other cultivars. Elevated *F*_0_ is widely interpreted as potentially associated with increased chlorophyll content or PSII reaction center damage [45]. However, the chlorophyll content of Dabaigu showed no significant difference from Changgu 4 and Zhonggu 19, while its *F_v_*/*F_m_* remained unchanged, collectively suggesting intact PSII functionality. Elevated *F*_0_ is more likely to originate from enhanced stability of light-harvesting pigment–protein complexes rather than structural damage within the photosystem. Furthermore, Zhangza 13 showed significantly higher *qN* than Zhonggu 19, indicating enhanced thermal dissipation capacity via the xanthophyll cycle to counteract high light stress [46]. This diversity in photoprotective mechanisms likely underpins cultivar-specific adaptation strategies to high-irradiation habitats in northern Shanxi.

### 3.5. Environmental Adaptation

The agro-pastoral ecotone in northern Shanxi Province experiences a semi-arid continental climate characterized by frequent high-temperature stress events. This study demonstrated superior performance in Dabaigu and Jingu 21 across key photosynthetic metrics including Pn, chlorophyll content, and *F_v_*/*F_m_*. These cultivars show robust light energy conversion efficiency and significant stress resilience potential. In terms of Pn, the high Pn of the two means that the unit leaf area can fix more carbon dioxide per unit time, which provides a sufficient material basis for dry matter accumulation [47]. The advantage of chlorophyll content ensures the ability of their leaves to capture and transform light energy [48]. The chlorophyll fluorescence parameter *F_v_*/*F_m_* was always maintained at a high level, indicating that the photosynthetic apparatus of these two varieties could maintain a stable functional state under high-temperature and drought stress, and the degree of damage was relatively light, which fully reflected that they had strong light energy conversion efficiency and potential ability to cope with adversity [49]. Compared with Dabaigu, Jingu 21 showed stronger adaptability to the arid environment, and its higher WUE made it have an obvious advantage in the water-scarce environment in the agro-pastoral ecotone of northern Shanxi, which effectively alleviated the inhibitory effect of drought stress on growth. The differential adaptation mechanism of the two provides an important physiological and ecological basis for the screening and promotion of foxtail millet varieties in this region. Compared with the performance of Dabaigu, Jinmiao K1 and Changgu 4 show a pronounced decline in photosynthetic performance under high-temperature conditions. Possible approaches to enhance stress resistance may involve exogenous regulation, such as foliar application of melatonin or silicon fertilizer [50].

## 4. Materials and Methods

**Figure 8:**
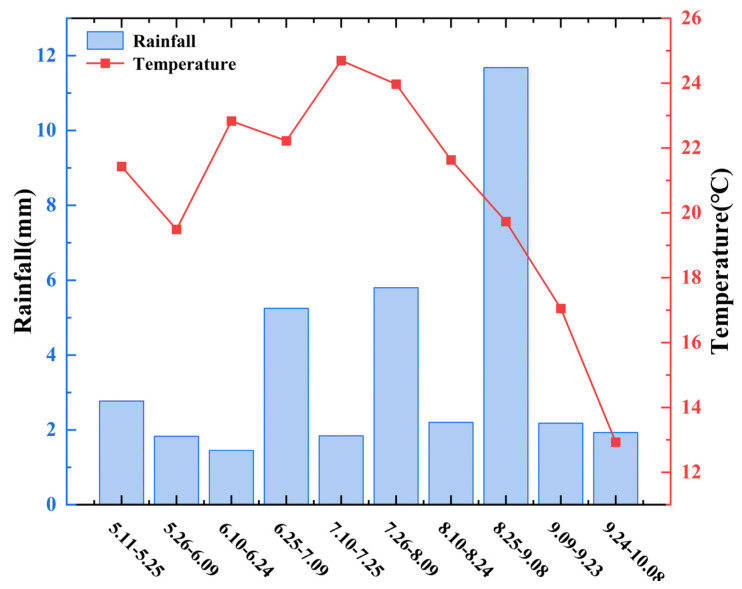
Temperature and precipitation during the whole growth period of foxtail millet in 2024.

The experiment was conducted from April to October 2024 in Shanyin County, Shuozhou City, Shanxi Province. The study site is located at an altitude of 1002 m with light sandy loam soil and a temperate continental climate featuring an average annual frost-free period of 130 days and mean annual precipitation of 410 mm [51]. The initial soil fertility characteristics at the experimental site are detailed in Table 4. The parameters of soil analysis were selected from the soil background value of field production. Temporal dynamics of precipitation and ambient temperature during the entire foxtail millet growth cycle (May–October) were aggregated at 15-day intervals (Figure 8).

### 4.1. Test Materials

The experimental materials consisted of six foxtail millet cultivars: Zhonggu 19, Zhangza 13, Jinmiao K1, Jingu 21, Changgu 4, and Dabaigu. We selected foxtail millet materials on the basis of their regional adaptability, genetic diversity and production application value. Detailed information on their origins is provided in Table 5.

### 4.2. Experimental Design

The experimental investigation employed a randomized complete block design with four replications to evaluate six newly released foxtail millet cultivars. A uniform planting geometry with 55 cm row spacing and 3 cm plant spacing was implemented consistently across all experimental units for each cultivar. Inter-seedling selection was carried out at the 3–4 leaf stage. The final plant density was maintained at 495,000 plants per hectare. Compound fertilizer (N-P_2_O_5_-K_2_O, the NPK nutrient content is 28-13-10) was applied at 600 kg per hectare.

### 4.3. Determination Indicators and Methods

#### 4.3.1. Determination of Agronomic Traits

Uniform foxtail millet plants were subjected to morphological characterization, with plant height measured from base to panicle tip using a straight ruler, and flag leaf length and width recorded with a measuring ruler. Stem diameter was determined with vernier calipers. The calculation formula for leaf area is as follows [52]

LA = L × W × 0.75(1)

where LA is the leaf area of a single leaf (cm^2^), L is the length of the leaf midrib (cm), W is the maximum width of the leaf (cm), and 0.75 is the leaf area coefficient.

#### 4.3.2. Determination of Leaf Photosynthetic Data

Ecophysiological determinants of photosynthesis were quantified under clear sky conditions (09:00–18:00) using a portable photosynthesis system (CI-340, CID Bio-Science Inc., USA). Measurements occurred at hourly intervals on fully expanded flag leaves from three pre-labeled uniformly growing plants per experimental plot. Photosynthetic parameters quantified included photosynthetically active radiation (PAR), net photosynthetic rate (Pn), transpiration rate (Tr), stomatal conductance (Gs), intercellular CO_2_ concentration (Ci), relative air humidity (RH), leaf temperature (Tl), and ambient temperature (Ta). To eliminate time errors, each replication determination was carried out randomly among the cultivars. Leaf water use efficiency (WUE) was calculated using the formula [53]

WUE = Pn/Tr(2)

#### 4.3.3. Determination of Chlorophyll Fluorescence Data

Chlorophyll fluorescence kinetics were quantified in dark-adapted leaves (30 min) using a portable pulse amplitude modulation fluorometer (PAM-2500, Walz, Effeltrich, Germany) under cloud-free conditions with saturating ambient photosynthetic photon flux density. Leaves from three representative plants per foxtail millet cultivar were selected on the basis of uniform growth vigor, optimal physiological status, and absence of disease or pest damage. The non-photochemical quenching (*qN*), initial fluorescence (*F*_0_), and maximum fluorescence (*F_m_*) of the second fully expanded leaves of foxtail millet were recorded, with subsequent derivation of variable fluorescence (*F_v_*), maximum photochemical efficiency (*F_v_*/*F_m_*), and potential activity (*F_v_*/*F*_0_) calculated [54].

*F_v_* = *F_m_* − *F*_0_ *F_v_*/*F_m_* = (*F_m_* − *F*_0_)/*F_m_* *F_v_*/*F*_0_ = (*F_m_* − *F*_0_)/*F*_0_ (3)

#### 4.3.4. Determination of SPAD in Leaves

Leaf chlorophyll content was quantified using a handheld chlorophyll meter (SPAD-502 Plus, Konica Minolta Sensing, Inc., Osaka, Japan). Chlorophyll content was measured in the penultimate functional leaves of foxtail millet plants, avoiding leaf veins, with 10 randomized samplings each at the proximal, medial, and distal leaf sections.

### 4.4. Data Processing

Data were processed using Microsoft Excel 2022 (Microsoft, Redmond, WA, USA) and IBM SPSS Statistics 25 software (SPSS Inc., Chicago, IL, USA). One-way ANOVA was used to test the overall differences in each trait among varieties. When the analysis of variance showed significant differences (*p* < 0.05), Duncan’s new multiple range test was used for post hoc pairwise comparison to determine the specific difference group. Statistical analyses were performed using Origin 2024 software (Origin Lab., Northampton, MA, USA). The data are presented in the form of mean ± s.e.m. For the principal component analysis (PCA) results, the data are visualized as a score plot, where each point represents an individual cultivar, with the position determined by its scores on the first two principal components (PC1 and PC2).

## 5. Conclusions

This study conducted a systematic comparison of photosynthetic physiological parameters and agronomic traits across six foxtail millet cultivars from the agro-pastoral ecotone of northern Shanxi Province. This investigation revealed significant variations in Pn, Gs, light energy utilization, and morphological characteristics across the foxtail millet cultivars. The results demonstrated that cultivars Dabaigu and Jingu 21 showed superior performance in key photosynthetic metrics, including Pn, chlorophyll content, and photochemical efficiency. However, Jinmiao K1 and Zhangza 13 showed inferior performance across multiple parameters. These findings deliver critical breeding references for foxtail millet cultivars in the agro-pastoral ecotone of northern Shanxi Province while providing robust scientific evidence for deciphering the intrinsic mechanisms governing photosynthetic physiology and environmental adaptation in this crop. In the future, molecular marker-assisted breeding can be carried out on the physiological mechanisms of these two varieties (such as stomatal regulation mode or the osmotic substance accumulation law) to further improve the adaptability of foxtail millet in semi-arid areas. Since we are currently conducting the second year of the experiment, only one year of experimental data is covered. We plan to integrate and analyze the comprehensive dataset after the completion of the second year of the experiment.

## Figures and Tables

**Table 1:** Leaf water use efficiency of different cultivars of foxtail millet.

Treatment	Average Pn	Average Tr	WUE
	(µmol·m^−2^·s^−1^)	(mmol·m^−2^·s^−1^)	(µmol·CO_2_/mmol·H_2_O)
Zhonggu 19	18.49 ± 0.24 c	2.38 ± 0.08 c	7.77 ± 0.18 a
Zhangza 13	16.29 ± 0.52 d	2.64 ± 0.03 bc	6.17 ± 0.24 b
Jinmiao K1	12.87 ± 0.16 e	2.40 ± 0.10 c	5.36 ± 0.15 c
Jingu 21	20.72 ± 0.31 b	2.72 ± 0.07 b	7.62 ± 0.26 a
Changgu 4	18.74 ± 0.14 c	2.95 ± 0.16 a	6.35 ± 0.28 b
Dabaigu	22.99 ± 0.23 a	2.86 ± 0.03 ab	8.04 ± 0.05 a

Note: Different lowercase letters after data in the same column indicate that there was a significant difference between the varieties at the *p* < 0.05 level (Duncan’s multiple comparison method based on one-way ANOVA).

**Table 2:** Daily average value of photosynthetic factors of different foxtail millet cultivars.

Treatment	Ci	Gs	RH	Tl
	(µmol·m^−2^·s^−1^)	(mmol·m^−2^·s^−1^)	(µmol·CO_2_/mmol·H_2_O)	°C
Zhonggu 19	196.43 ± 2.05 c	92.94 ± 3.83 c	38.33 ± 0.15 a	35.08 ± 0.91 b
Zhangza 13	191.76 ± 0.85 c	132.33 ± 3.01 a	33.38 ± 0.09 c	35.86 ± 0.70 b
Jinmiao K1	182.95 ± 1.70 d	74.96 ± 1.83 d	34.21 ± 0.06 bc	36.14 ± 0.80 ab
Jingu 21	206.80 ± 2.05 b	111.30 ± 8.30 b	35.50 ± 0.24 bc	37.51 ± 0.92 ab
Changgu 4	230.42 ± 4.12 a	104.83 ± 7.76 bc	37.27 ± 0.05 a	36.89 ± 0.82 ab
Dabaigu	211.55 ± 2.80 b	127.90 ± 2.61 a	36.74 ± 0.36 b	38.08 ± 1.05 a

Note: Different lowercase letters after data in the same column indicate that there was a significant difference between the varieties at the *p* < 0.05 level (Duncan’s multiple comparison method based on one-way ANOVA).

**Table 3:** The eigenvalues of each principal component.

Principal Component Number	Eigenvalue	Percentage of Variance (%)	Cumulative (%)
1	5.99356	37.45978	37.45978
2	2.99472	18.717	56.17678
3	2.42137	15.13353	71.31031
4	1.93217	12.07604	83.38635

Note: Eigenvalues, variance contributions, and cumulative variance of the principal components from PCA. Proportion of total phenotypic variance explained by a single principal component. PC1 accounts for 37.5% of total variance, representing the most significant trait differentiation. Accumulated variance is explained by successive principal components. The first two components (PC1 + PC2) collectively explain 56.2% of total variance.

**Table 4:** Pedochemical baseline indicators in the 0–20 cm plough layer before the 2024 sowing season.

Total P	Total K	Total N	Available K	Available N	Available P	Organic Matter	PH
(g/kg)	(g/kg)	(g/kg)	(mg/kg)	(mg/kg)	(mg/kg)	(g/kg)	Value
0.59	15.50	0.62	89.00	69.10	21.70	11.20	9.04

**Table 5:** Origins of foxtail millet cultivars.

Cultivar	Origin
Zhonggu 19	Institute of Crop Sciences, Chinese Academy of Agricultural Sciences
Zhangza 13	Zhangjiakou Academy of Agricultural Sciences
Jinmiao K1	Chifeng Agriculture and Animal Husbandry Science Research Institute
Jingu 21	Economic Crops Research Institute, Shanxi Agricultural University
Changgu 4	Foxtail Millet Research Institute, Shanxi Agricultural University
Dabaigu	Guangling, local variety

## Data Availability

The data that support this study are available upon reasonable request from the corresponding author.
